# Systemic antibiotics increase microbiota pathogenicity and oral bone loss

**DOI:** 10.1038/s41368-022-00212-1

**Published:** 2023-01-12

**Authors:** Xulei Yuan, Fuyuan Zhou, He Wang, Xinxin Xu, Shihan Xu, Chuangwei Zhang, Yanan Zhang, Miao Lu, Yang Zhang, Mengjiao Zhou, Han Li, Ximu Zhang, Tingwei Zhang, Jinlin Song

**Affiliations:** Chongqing Key Laboratory for Oral Diseases and Biomedical Sciences, Chongqing Municipal Key Laboratory for Oral Biomedical Engineering of Higher Education, Stomatological Hospital of Chongqing Medical University, Chongqing, China

**Keywords:** Microbial communities, Periodontitis

## Abstract

Periodontitis is the most widespread oral disease and is closely related to the oral microbiota. The oral microbiota is adversely affected by some pharmacologic treatments. Systemic antibiotics are widely used for infectious diseases but can lead to gut dysbiosis, causing negative effects on the human body. Whether systemic antibiotic-induced gut dysbiosis can affect the oral microbiota or even periodontitis has not yet been addressed. In this research, mice were exposed to drinking water containing a cocktail of four antibiotics to explore how systemic antibiotics affect microbiota pathogenicity and oral bone loss. The results demonstrated, for the first time, that gut dysbiosis caused by long-term use of antibiotics can disturb the oral microbiota and aggravate periodontitis. Moreover, the expression of cytokines related to Th17 was increased while transcription factors and cytokines related to Treg were decreased in the periodontal tissue. Fecal microbiota transplantation with normal mice feces restored the gut microbiota and barrier, decreased the pathogenicity of the oral microbiota, reversed the Th17/Treg imbalance in periodontal tissue, and alleviated alveolar bone loss. This study highlights the potential adverse effects of long-term systemic antibiotics-induced gut dysbiosis on the oral microbiota and periodontitis. A Th17/Treg imbalance might be related to this relationship. Importantly, these results reveal that the periodontal condition of patients should be assessed regularly when using systemic antibiotics in clinical practice.

## Introduction

Periodontitis, affecting approximately half the worldwide adult population, is a widespread oral disease that causes disruption of the periodontal soft and hard tissue. Several systemic diseases are connected with periodontitis, such as obesity, diabetes, arthritis, heart disease, and cancer. Treatment of systemic diseases with pharmacotherapy has been found to have adverse effects on the periodontal tissue and can even aggravate periodontitis. For example, long-term use of phenytoin, cyclosporin, or calcium channel blockers leads to drug-induced gingival hyperplasia.^1^ Dexamethasone, used for arthritic joint treatment, increases alveolar bone loss.^2^ Chemotherapy for cancer also increases the susceptibility to periodontitis.^3^

Systemic antibiotic treatment is widely used in several infectious diseases, such as chronic osteomyelitis, bacterial endophthalmitis, airway infection, and acne. However, the use of antibiotics can cause gut dysbiosis.^4,5^ The intestinal microbiota is the most diverse and largest bacterial population in the human body, contributing to physiological development, nutrient digestion, and defense against the colonization of pathogens.^6^ Long-term use of systemic antibiotics disturb the gut microbiota by decreasing the diversity, changing the metabolome, damaging the intestinal defense, and causing antibiotic resistance.^4,7^ Gut dysbiosis leads to increased intestinal permeability, which allows pathogen and microbial products to cause endotoxemia, in turn affecting distant organs through hematogenous spread.^8^ Gut dysbiosis caused by antibiotics is associated with various systemic diseases and some of those diseases are also correlated with periodontitis.^9–12^ Thus, the gut microbiota might be a potential link between general health and periodontitis.^8^ However, whether gut dysbiosis caused by pharmacotherapy with antibiotics negatively affects periodontitis remains unclear.

On the other hand, systemic antibiotic treatment itself is an adjunctive therapy for periodontitis. Unlike local antibiotic treatment, systemic antibiotic treatment can better expose antibiotics to pathogens widely distributed in the mouth, such as pathogens in the dorsum of the tongue and tonsil crypts.^13^ Compared with scaling and root planning alone, systemic antibiotic treatment combined with scaling and root planning provides better clinical results, including more probing pocket depth reductions and clinical attachment gains.^14^ Nevertheless, the adverse impacts of systemic antibiotics on the intestinal microbiota and many other diseases cannot be ignored. The gut microbiota is linked with the oral microbiota via the digestive tract and some studies suggest that they are closely related. An abnormal oral microbiota leads to the translocation of oral pathogens to the gut, causing colon inflammation by activating the inflammasome.^15^ Compared to patients with similar clinical periodontal parameters but a healthy gut, patients with gut disease have more pathogenic bacteria in subgingival sites.^16^ The abnormal oral microbiota in turn actives the corresponding immune and inflammatory responses of the host, and contributes to periodontitis.^17^ Therefore, whether gut dysbiosis caused by antibiotics alters the oral microbiota and the responses of the host, thereby affecting periodontitis, is well worth exploring.

Several studies have found that a Th17/Treg (T-helper and regulatory T cells) imbalance is related to periodontitis. Th17 cells can secrete interleukin (IL)-17A, which promotes the production of other inflammatory cytokines such as IL-6 and RANKL, resulting in alveolar bone loss.^18,19^ On the contrary, Treg cells, a subset of CD4 + T lymphocytes, maintain immune homeostasis and protect tissue against inflammatory destruction in periodontitis.^20,21^ Foxp3 is a transcription factor specifically expressed by Treg cells; it also produces anti-inflammatory cytokines such as IL-10 and TGF-β.^18,22^ Dysbiosis of the gut microbiota in ovariectomized (OVX) rats aggravates periodontitis through a Th17/Treg imbalance, while probiotics or berberine can improve the gut microbiota and reverse the Th17/Treg imbalance, reducing alveolar bone loss.^23^ These studies indicate that a Th17/Treg imbalance might aggravate periodontitis and might be linked to the microbiota.

In this study, to reveal how systemic antibiotics affect the microbial pathogenicity of the oral-gut axis, mice were exposed to drinking water containing a cocktail of four antibiotics for four weeks to simulate long-term use of systemic antibiotics. A ligature-induced mouse model of periodontitis was used to study the subsequent influences on periodontitis. Moreover, fecal microbiota transplantation (FMT) was used to restore the gut microbiota disturbed by antibiotics. The research indicated that gut dysbiosis caused by antibiotics damages the gut barrier, disturbs the oral microbiota, and aggravates alveolar bone loss in periodontitis through a Th17/Treg imbalance. These findings highlight the need to assess the periodontal condition of patients who use systemic antibiotics.

## Results

### Long-term use of antibiotics caused gut dysbiosis and increased periodontitis-related pathogens in the oral microbiota

**Fig. 1:**
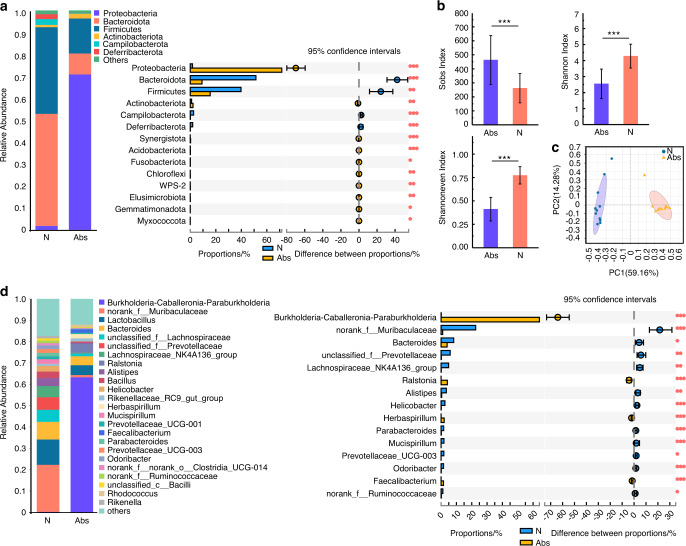
Long-term use of antibiotics caused gut dysbiosis. Community bar plot and Wilcoxon rank-sum test bar plot of the gut microbiota at the phylum (**a**) and genus level (**d**). Alpha diversity of the gut microbiota (**b**). PCoA analysis of the gut microbiota (**c**). *P < 0.05, **P < 0.01, ***P < 0.001

Mice in the Abs group were treated with a four-antibiotic cocktail (cefoxitin, gentamicin, metronidazole, and vancomycin, 1 mg·mL^−1^ of each) in their drinking water while mice in the N group received normal drinking water; both treatments lasted for four weeks (Fig S1a). The results indicated that the community evenness and community diversity of the gut microbiota in the Abs group were lower than those in the N group after antibiotic treatment (Fig. 1b). In the principal coordinate analysis (PCoA), the N and Abs groups were significantly distinguished on the genus level, suggesting differences in community composition between these groups (Fig. 1c). At the phylum level, the abundance of *Proteobacteria*, a potential factor in gut microbiota dysbiosis and gut disease,^24^ was sharply increased in the Abs group (Fig. 1a). At the genus level, the probiotics *Lachnospiraceae_NK4A136_group* and *Alistipes*^25^ were decreased in the Abs group (Fig. 1d, S2b).

**Fig. 2:**
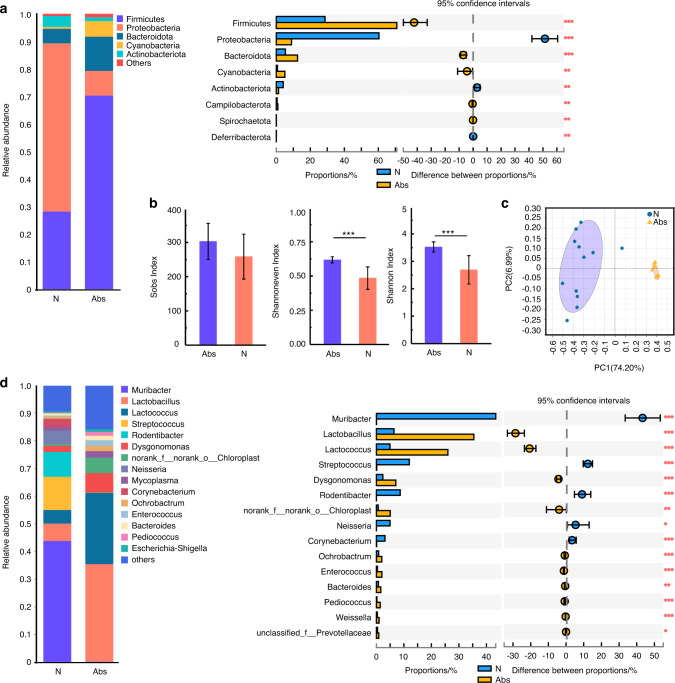
Long-term use of antibiotics increased periodontitis-related pathogens in the oral microbiota. Community bar plot and Wilcoxon rank-sum test bar plot of the oral microbiota at the phylum (**a**) and genus level (**d**). Alpha diversity of the oral microbiota (**b**). PCoA analysis of the oral microbiota (**c**). *P < 0.05, **P < 0.01, ***P < 0.001

Different from the gut microbiota results, antibiotics increased the community evenness and diversity of the oral microbiota (Fig. 2b). Antibiotics also changed the composition (Fig. 2a, c–d, S2c, d) and increased the pathogenicity of the oral microbiota. Bacteria associated with periodontal health were decreased, including *Streptococcus*, *Neisseria*, and *Corynebacterium*^26^ (Fig. 2d). Moreover, bacteria associated with periodontitis were increased, such as *Enterococcus* and *Dysgnomonas*^27^ (Fig. 2d). These results demonstrate that long-term use of antibiotics causes gut dysbiosis, increases periodontitis-related pathogens, and decreases periodontal health-related probiotics in the oral microbiota.

### Gut microbiota dysbiosis did not recover and oral pathogenicity in mice with experimental periodontitis increased two weeks after withdrawal of antibiotics

**Fig. 3:**
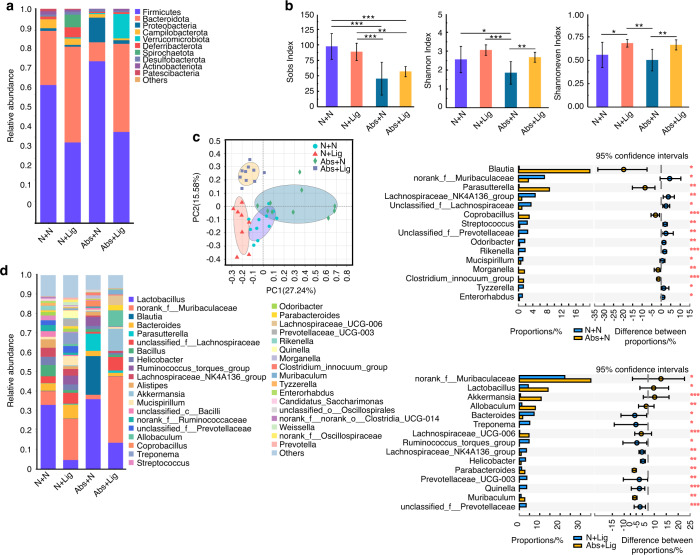
Gut microbiota dysbiosis did not recover after 2-week withdrawal of antibiotics. Community bar plot and Wilcoxon rank-sum test bar plot of the gut microbiota at the phylum (**a**) and genus level (**d**). Alpha diversity of the gut microbiota (**b**). PCoA analysis of the gut microbiota (**c**) *P < 0.05, **P < 0.01, ***P < 0.001

After four weeks, the antibiotic water was removed from the Abs group and then mice in the N and Abs groups were divided into two groups, respectively (*n* = 12): N + N group, N + Lig (Ligature) group, Abs+N group, and Abs+Lig group. An experimental periodontitis model was established in the N + Lig and Abs+Lig groups using a silk ligature for two weeks (Fig. S1a). As for the gut microbiota, the Sobs index revealed that 2 weeks after withdrawal of the antibiotics, the species richness in the Abs+N group was sharply decreased compared with the N + N group (Fig. 3b). Previous reductions in community evenness and community diversity caused by antibiotics (Abs+N and Abs+Lig groups) did not recover. It was worth mentioning that in the ligature groups (N + Lig and Abs+Lig), community evenness and community diversity were increased (Fig. 3b). Both antibiotics and periodontitis changed the community composition (Fig. 3c, S3a, b). In the Abs+N group, *Proteobacteria*, a potential factor in gut dysbiosis, were still abundant while the abundance of *Actinobacteriota* was significantly decreased (Fig. 3a, S4a). *Actinobacteriota* plays a vital role in modulating gut permeability, the immune system, metabolism, and the gut-brain axis.^28^ At the genus level, pathogenic bacteria were increased significantly, including *Blautia*, *Parasutterella*, and *Moganella*^29^ (Fig. 3d, S3b). However, the abundance of the gut health-related bacteria *norank_Muribaculaceae*^30^ was decreased in the Abs+N group (Fig. 3d, S3b). These data suggest that the gut microbiota could not recover after a 2-week withdrawal of antibiotics. A previous study also reported that antibiotics can disturb the gut microbiota with long-lasting effects.^31^

**Fig. 4:**
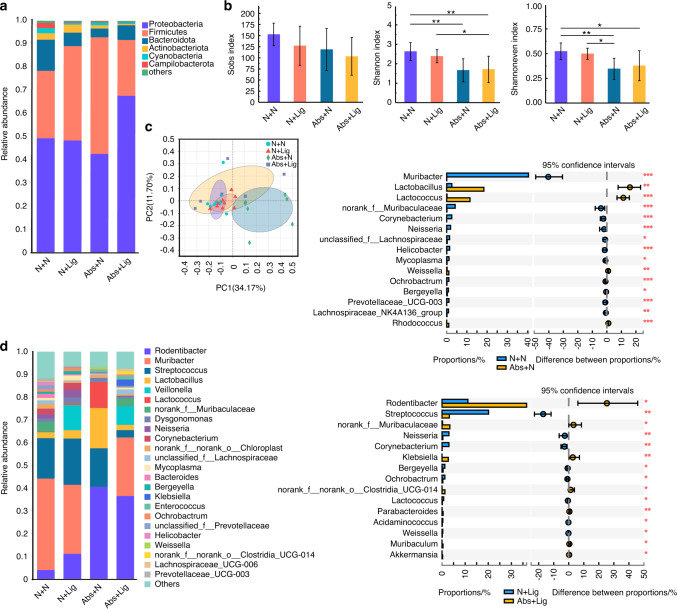
Oral pathogenicity in mice with experimental periodontitis increased after 2-week withdrawal of antibiotics. Community bar plot and Wilcoxon rank-sum test bar plot of the oral microbiota at the phylum (**a**) and genus level (**d**). Alpha diversity of the oral microbiota (**b**). PCoA analysis of the oral microbiota (**c**). *P < 0.05, **P < 0.01, ***P < 0.001

In the oral microbiota, although no significant differences in species richness were found between the different groups, the community evenness and community diversity were much lower in the antibiotic groups (Abs+N and Abs+Lig) compared to the groups without antibiotics (N + N, N + Lig) (Fig. 4b). In the groups with experimental periodontitis (N + Lig and Abs+Lig), antibiotic treatment increased the pathogenicity of the oral microbiota. Compared with the N + Lig group, the abundance of probiotics such as *Streptococcus*, *Neisseria*, *Bergeyella*, *Lactococcus*, and *Weissella* were significantly decreased, and the abundance of the oral pathological bacteria *Klebsiella* was increased in the Abs+Lig group^14,26,32^ (Fig. 4d, S3d). The results above reveal that, under the condition of periodontitis, antibiotic treatment can increase the pathogenicity of the oral microbiota.

### Antibiotics led to intestinal damage and aggravated alveolar bone loss

**Fig. 5:**
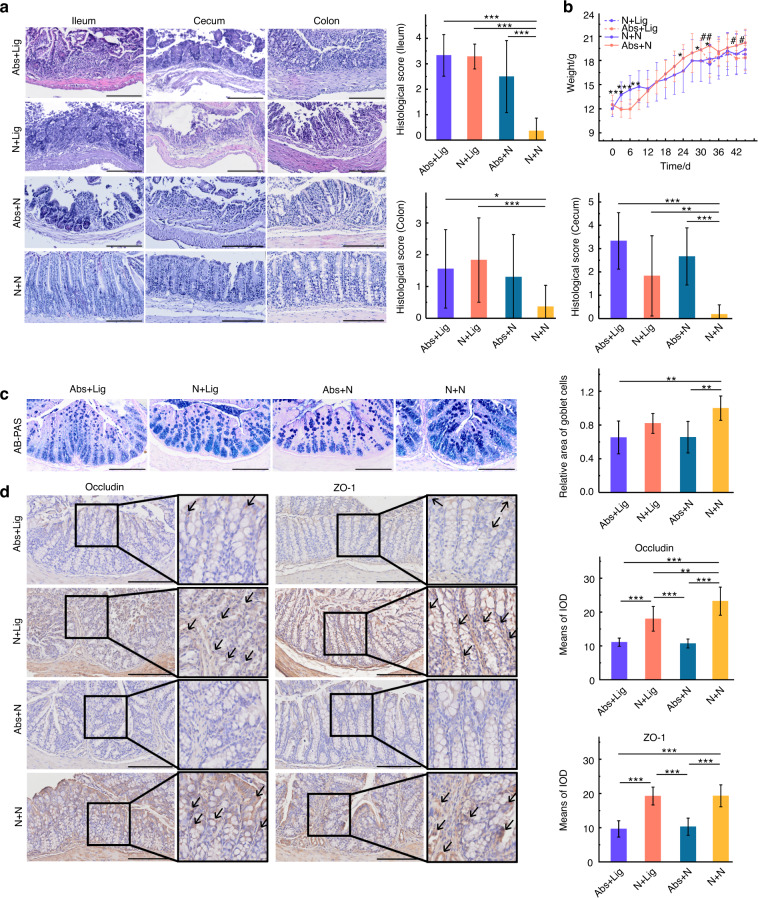
Antibiotics use led to intestinal damage. **a** HE staining and histological scores of the ileum, cecum, and colon (Scale bar = 200 μm). **b** Weight record of mice (*Abs+N vs N + N, ^#^Abs+N vs Abs+Lig). **c** AB-PAS staining and relative area of goblet cells of the colon (Scale bar = 200 μm). **d** Immunohistochemical analysis of occluding and ZO-1 positive area (Scale bar = 250 μm, ROI: 80 μm × 80 μm). *P < 0.05, **P < 0.01, ***P < 0.001

**Fig. 6:**
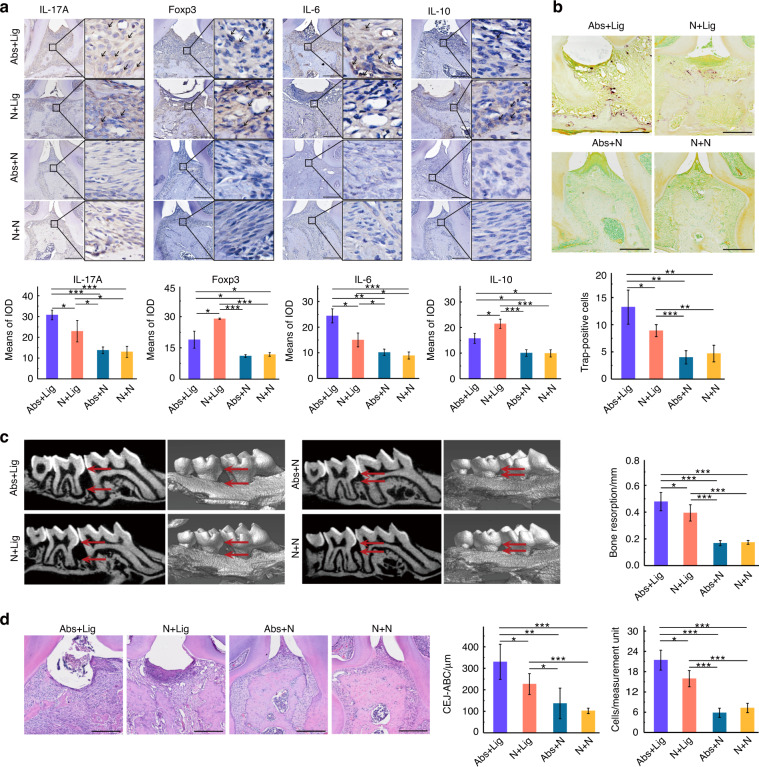
Antibiotics use aggravated alveolar bone loss. **a** Immunohistochemical analysis of IL-17A, IL-6, Foxp3, and IL-10 (Scale bar = 250 μm, ROI: 80 μm × 80 μm). **b** Trap staining and analysis (Scale bar = 250 μm). **c** Micro-CT analysis. **d** HE staining of maxillae (Scale bar = 250 μm), CEJ-ABC measurement, and neutrophil count analysis. *P < 0.05, **P < 0.01, ***P < 0.001

The body weights of mice were recorded throughout the experiment. An obvious reduction in body weight was observed in the Abs group from D1 to D9. Later, the weights of these mice increased rapidly to a level higher than that of mice in the N group. After D30, Mice with ligatures (N + Lig, Abs+Lig) exhibited significant reductions in body weight, especially in the Abs+Lig group (Fig. 5b). Obvious intestinal damage was observed in the groups administered antibiotics (Abs+N and Abs+Lig), especially in the ileum and cecum, as evidenced by hematoxylin-eosin (HE) staining and histological score analysis (Fig. 5a). Less goblet cells (Fig. 5c) and less positive expression of tight junction-related proteins (Fig. 5d) were observed in the groups administered antibiotics (Abs+N and Abs+Lig), as compared to the groups without antibiotics. Moreover, antibiotics aggravated periodontitis in mice with ligatures. According to the micro-CT and HE staining analyses, the Abs+Lig group exhibited greater alveolar bone loss and neutrophil infiltration than the N + Lig group (Fig. 6c, d). In addition, there were more TRAP-positive osteoclasts and greater expression of Th17 cell-related proinflammatory cytokines (IL-17A, IL-6) in the Abs+Lig group than in the N + Lig group (Fig. 6a, b). Antibiotics also decreased the expression of Treg cell-related proinflammatory cytokines (Foxp3 and IL-10) in the Abs+Lig group compared with the N + Lig group (Fig. 6a).

### FMT with normal mice feces improved the gut dysbiosis caused by antibiotics but had no obvious effect on the oral microbiota

FMT, a new form of treatment, rebuilds the species composition and physiological function of the normal gut microbiota by transplanting the fecal microbiota of healthy donors to diseased recipients. Another 30 mice were provided with the four-antibiotic cocktail in drinking water for four weeks, the same as described above for the Abs group. Then, the antibiotic water was replaced with regular drinking water and the mice were divided into FMT-N and FMT-Abs groups. The fecal microbiota of mice in the N + N and Abs+N groups were transferred to the FMT-N and FMT-Abs mice, respectively (Fig. S1b). Two weeks after FMT, the gut microbiota of the recipient mice in the FMT-N and FMT-Abs groups were clearly distinguished (Fig. S5a. c, d, S7a, b). There were more *Bacteroidota* and *Actinobacteriota* and fewer *Firmicutes* in the gut microbiota of the FMT-N group compared to the FMT-Abs group (Fig. S5a). The recipient mice in each group showed similar gut microbiota to the donor mice, which indicates the success of FMT. Although the composition of the oral microbiota was different (Fig. S6a, c, d, S7c, d), there were no statistically significant differences in alpha and beta diversity between the FMT-Abs and FMT-N groups (Fig. S6b, c). These results indicate that FMT did not directly alter the oral microbiota as it did the gut microbiota.

### The pathogenicity of the oral microbiota in antibiotic-treated experimental periodontitis mice decreased with FMT of normal mice feces

**Fig. 7:**
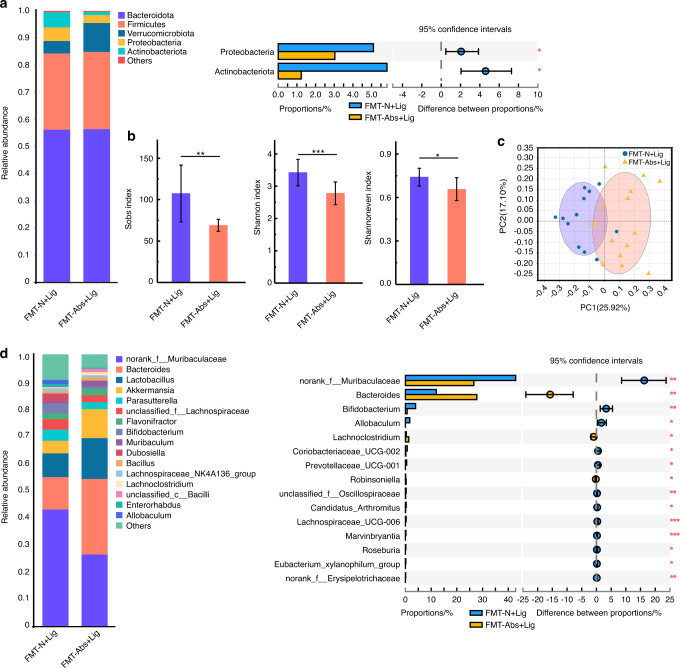
FMT with normal mice feces partially restored the gut microbiota disturbed by antibiotics. Community bar plot and Wilcoxon rank-sum test bar plot of the gut microbiota at the phylum (**a**) and genus level (**d**). Alpha diversity of the gut microbiota (**b**). PCoA analysis of the gut microbiota (**c**). *P < 0.05, **P < 0.01, ***P < 0.001

**Fig. 8:**
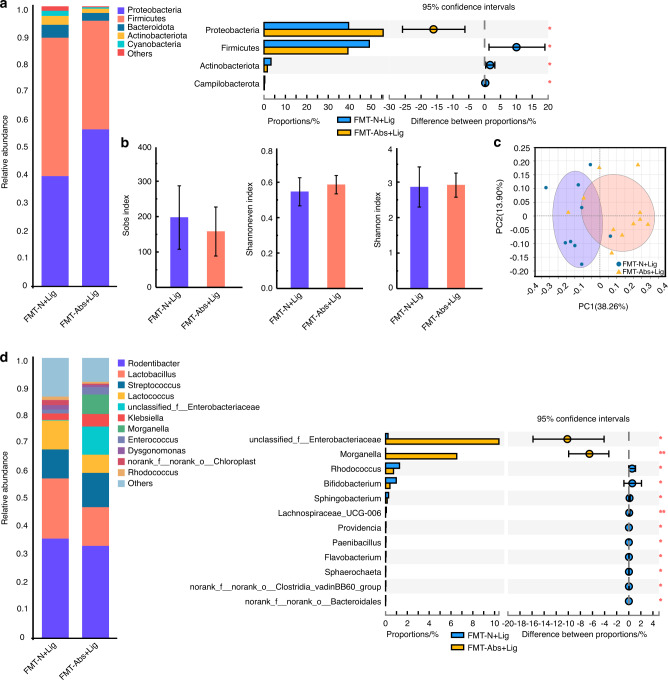
FMT with normal mice feces reduced the pathogenicity of the oral microbiota in antibiotic-treated experimental periodontitis mice. Community bar plot and Wilcoxon rank-sum test bar plot of the oral microbiota at the phylum (**a**) and genus level (**d**). Alpha diversity of the oral microbiota (**b**). PCoA analysis of the oral microbiota (**c**). *P < 0.05, **P < 0.01, ***P < 0.001

Two weeks after FMT, the experimental periodontitis model was established in all mice (FMT-N + Lig and FMT-Abs+Lig groups) (Fig. S1b). In the FMT-N + Lig group, the gut microbiota of mice showed higher alpha diversity, related to a more stable and healthier microbiota, than mice in the FMT-Abs+Lig group (Fig. 7b). The abundances of probiotics, such as *norank_Muribaculaceae* and *Prevotellaceae_UCG-001*,^30^ were much higher in the FMT-N + Lig group (Fig. 7d, S8b). As for the oral microbiota, although no obvious difference in alpha diversity was found between the FMT-Abs+Lig and FMT-N + Lig groups, there was a statistically significant difference in beta diversity (Fig. 8b, c). At the genus level, there was a lower abundance of the oral pathological bacteria *unclassified_Enterobacteriaceae*^15^ and the opportunistic pathogen *Morganella*^33^ in the FMT-N + Lig group compared to the FMT-Abs+Lig group (Fig. 8d, S8d). These results showed when experimental periodontitis was induced, a healthier oral microbiota composition was formed in mice with FMT of normal mice feces.

### FMT of normal mice feces alleviated intestinal damage and alveolar bone loss in mice with experimental periodontitis

**Fig. 9:**
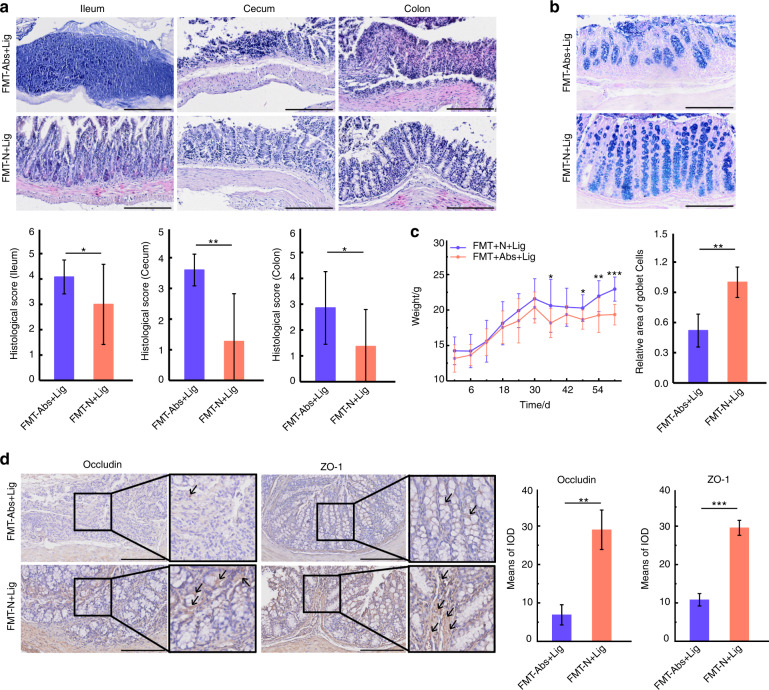
FMT of normal mice feces alleviated intestinal damage. **a** HE staining and histological scores of the ileum, cecum, and colon (Scale bar = 200 μm). **b** AB-PAS staining and relative area of goblet cells of the colon (Scale bar = 200 μm). **c** Weight record of mice. **d** Immunohistochemical analysis of occludin and ZO-1 (Scale bar = 200 μm, ROI: 200 μm × 200 μm). *P < 0.05, **P < 0.01, ***P < 0.001

**Fig. 10:**
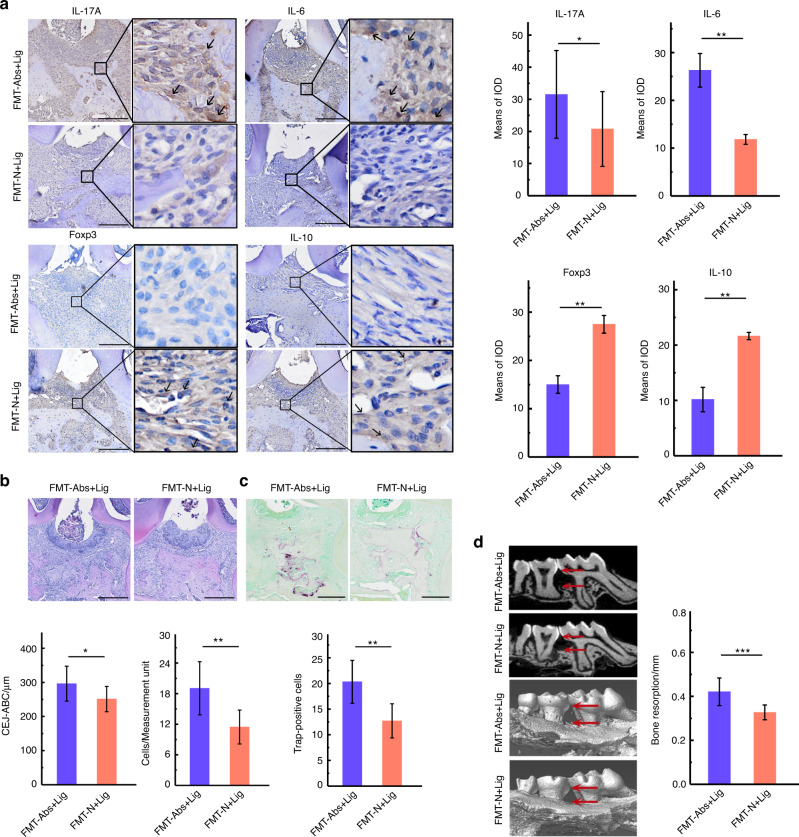
FMT of normal mice feces alleviated alveolar bone loss in mice with experimental periodontitis. **a** Immunohistochemical analysis of IL-17A, IL-6, Foxp3, and IL-10 (Scale bar = 250 μm, ROI: 80 μm×80 μm). **b** HE staining of maxillae (Scale bar = 250μm), CEJ-ABC measurement and neutrophil count analysis. **c** Trap staining and analysis (Scale bar = 250 μm). **d** Micro-CT analysis. *P < 0.05, **P < 0.01, ***P < 0.001

The body weights of the mice were recorded throughout the experiment. After D30, mice in the FMT-N group exhibited greater weight gains than mice in the FMT-Abs group. After ligature placement, the weight difference between the FMT-N + Lig and FMT-Abs+Lig groups became even larger (Fig. 9c). With regard to intestinal damage, the FMT-N + Lig group exhibited lower histological scores, a larger area of goblet cells and more positive expression of tight junction proteins than the FMT-Abs+Lig group (Fig. 9a, b, d). This is probably because FMT of normal mice feces rebuilt the antibiotic-disrupted gut microbiota and reduced its pathogenicity. Even so, pathological damage was still observed in the gut tissue, which could be explained by the adverse effects of four weeks of antibiotics use. Additionally, alveolar bone loss, neutrophil infiltration, and TRAP-positive osteoclasts were much lower in the FMT-N + Lig group than in the FMT-Abs+Lig group (Fig. 10b–d). Immunohistochemical staining revealed increased expression of Treg-related transcription factors and cytokines (Foxp3, IL-10) and reduced expression of Th17-related cytokines (IL-17A, IL-6) (Fig. 10a) in the FMT-N + Lig group compared to the FMT-Abs+Lig group. These results indicate that FMT of normal mice feces alleviated intestinal damage and periodontitis-induced alveolar bone loss in mice administered antibiotics.

## Discussion

The gut microbiota and oral microbiota are the two largest and most diverse bacterial populations in the human body. Periodontitis is a common oral inflammatory disease associated with disruption of the oral microbiota. Periodontitis-associated abnormal oral microbiota can affect the gut microbiota and gut diseases.^34^ Abnormal gut microbiota might also have the potential to affect the oral microbiota and aggravate periodontitis.^35^ Mice in this study were administered antibiotics for four weeks, and antibiotic administration led to decreased diversity and obvious dysbiosis in the gut microbiota, consistent with previous reports. Interestingly, the community richness of the gut microbiota in the Abs group was increased compared to the N group. This may be because the total microbial load might increase after the use of antibiotics due to the proliferation of antibiotic-resistant bacteria.^36^ After inducing experimental periodontitis, alveolar bone resorption was aggravated in mice administered antibiotics, compared with mice receiving no antibiotic treatment. While many studies, including animal experiments and clinical trials in humans, have used systemic antibiotic treatment as adjunctive therapy for periodontitis, resulting in reduced alveolar bone loss or enhanced treatment effects,^14,17^ the current study focused on whether long-term systemic antibiotic use has adverse effects on periodontitis due to the resulting microbiota dysbiosis. Mice were provided with antibiotic drinking water containing cefoxitin, gentamicin, metronidazole, and vancomycin (1 mg/ml of each) for four weeks. The types, doses, and duration of antibiotics were different from previous studies. Normal gut microbiota plays a vital role in maintaining the structural integrity of the gut barrier. Antibiotics can disrupt the normal gut microbiota, decreasing the expression of tight junction proteins and the apoptosis of intestinal epithelial cells. This disruption might lead to inflammation or damage to the intestinal barrier, including epithelial tissue damage and cecal swelling.^37^ Severe damage to the intestinal structure and decreased tight junction protein expression were observed in the groups administered antibiotics in the current study, which suggests that four weeks of exposure to antibiotic drinking water destroyed the intestinal barrier. Destruction of the intestinal barrier might increase gut permeability leading to increased pathogens and microbial products in circulation, which then affects distant tissue such as periodontal tissue. On the contrary, protecting the intestinal barrier might improve bone loss caused by antibiotics. One study reported that a probiotic or MDY-1001 (a high-molecular-weight polymer) can prevent post-antibiotic femur resorption by improving gut dysbiosis and protecting the gut barrier from disruption.^38^ Our findings demonstrate that increased alveolar bone loss might be related to disruption of the intestinal barrier after antibiotic-induced gut dysbiosis.

The pathogenicity of the oral microbiota in mice administered antibiotics was higher than that of mice with no antibiotic treatment after experimental periodontitis. This suggests that under a state of periodontitis, gut dysbiosis caused by antibiotics can affect the oral microbiota. However, the oral cavity was inevitably exposed to antibiotics when mice drank the antibiotic water, which may have affected the oral microbiota to some extent. Therefore, FMT was performed, directly affecting the gut microbiota without contact with the oral microbiota, in order to restore the disturbed gut microbiota in mice administered antibiotics. When experimental periodontitis was induced, mice that received FMT with normal mice feces showed lower pathogenicity of the oral microbiota and less alveolar bone loss. The possible explanation for this finding is as follows. First, healthy gut microbiota can stimulate the immune system to inhibit inflammation and maintain gut homeostasis. Gut dysbiosis caused by antibiotics might break gut homeostasis and allow bacteria or their metabolites to enter the bloodstream, in turn, causing systemic inflammation.^39–41^ Systemic inflammation further influences the local inflammatory environment, as observed in the Abs+Lig group where there was increased expression of inflammatory factors and reduced expression of anti-inflammatory factors in the periodontal tissue. The inflammatory periodontal microenvironment then facilitated the reproduction of periodontitis-related bacteria and increased periodontal tissue destruction.^42,43^ FMT from healthy donor mice partially reconstructed the disturbed gut microbiota caused by antibiotics and might ameliorate the systemic inflammation and local inflammatory periodontal microenvironment described above.

In periodontal lesions, Th17 cell-related cytokines (IL-17A, IL-6, IL-23, and IL-21) and osteoclastogenic mediators (RANKL) are significantly increased.^44^ Treg cells are an important subpopulation of immunosuppressive CD4 + T cells; they secrete anti-inflammatory cytokines (IL-10, IL-4, TGF-β) and downregulate RANKL expression, inhibiting alveolar bone resorption in periodontitis.^45^ Th17 cells and Treg cells have opposite immune regulation functions on bone metabolism. In this study, after ligature, mice with gut dysbiosis caused by antibiotics exhibited greater alveolar bone resorption and more osteoclasts compared with ligature-only mice. Greater expression of IL-17A and IL-6 and less expression of Foxp3 and IL-10 were observed in periodontal tissue. FMT of normal mice feces restored the gut microbiota of mice exposed to antibiotic treatment and reversed the expression of Th17-related factors and Treg-related factors in periodontal tissue. These results suggest that under a state of periodontitis, gut dysbiosis caused by antibiotics might induce Th17/Treg imbalance in periodontal tissue leading to alveolar bone loss. Rebuilding the gut microbiota reversed the Th17/Treg imbalance and alleviated periodontitis. In addition, antibiotic treatment created an inflammatory periodontal microenvironment with more pro-inflammatory factors and anti-inflammatory factors. Periodontitis is characterized by a bidirectional imbalance between the inflammatory response and microbiota. The abnormal microbiota also results in host response and uncontrolled inflammation.^17^ Our findings suggest that antibiotic use caused a periodontal inflammatory microenvironment and increased the abundance of bacteria associated with periodontitis in the oral microbiota, further aggravating alveolar bone loss. In this study, immunohistochemical staining was used to detect Th17-related and Treg-related cytokines in periodontal tissues. To more accurately explore immune cell changes, the use of flow cytometry or high-throughput sequencing is recommended in future studies.

Interestingly, mice with only ligatures (N + Lig group) also showed higher histological scores than mice without any treatment. How periodontitis causes this intestinal damage may be explained by the effect of experimental periodontitis on systemic inflammation. It has been reported that experimental periodontitis leads to high levels of inflammatory cytokines in plasma and increased monocytes in the bone marrow.^46^ The systemic inflammation caused by ligature-induced periodontitis may cause further intestinal damage in mice with periodontitis only. The transfer of periodontal pathogenic bacteria from the oral cavity to the gut may also be one of the reasons underlying intestinal barrier damage in mice administered antibiotics and induced periodontitis. However, debate remains as to whether oral bacteria can reach and colonize the gut. The healthy gut microbiota has colonization resistance, which prevents the invasion of alien bacteria.^24^ Moreover, researchers have found that the oral and gut microbiota are completely distinct in healthy adults.^47^ However, other studies have reported that colonization resistance may be destroyed and oral to-gut bacterial colonization may be possible when there is an unhealthy gut microbiota. For example, ampicillin treatment can result in the gut colonization of oral Klebsiella spp.^48^ Similarly, in the presence of inflammatory bowel disease, the oral pathogenic bacteria Klebsiella and Enterobacter proliferate abnormally and colonize the gut, aggravating gut inflammation.^15^ Therefore, we hypothesized that systemic inflammation caused by experimental periodontitis and ectopic colonization of the oral microbiota might be some of the mechanisms by which periodontitis affects the gut in mice administered antibiotics. The specific mechanisms need to be explored in future studies.

On the other hand, it must be acknowledged that the antibiotic treatment in our study involved exposure to compound broad-spectrum antibiotic drinking water for four weeks, which is not exactly the same as a human clinical treatment. Moreover, compared with 16S rRNA gene sequencing used in the study, shotgun next-generation sequencing metagenomics has several advantages, including the ability to more comprehensively characterize microbiome complexity and identify more species in each sample.^49^ Better animal models and detection methods should be used for in-depth investigations. In addition, except for the local inflammatory changes in the periodontal tissue, the systemic inflammatory responses should also be explored in the future.

On the whole, this research demonstrates that systemic antibiotics induce gut dysbiosis, leading to a Th17/Treg imbalance in periodontal tissue. The findings emphasize the potential adverse effects of systemic antibiotics on the oral microbiota and periodontitis. When systemic antibiotics are used clinically, assessment of the patient’s periodontal condition is important, especially for patients who already have periodontitis.

## Materials and methods

### Animal experiment

Four-week-old male C57 mice were raised in an SPF environment in the key laboratory of the Stomatological Hospital of Chongqing Medical University. They were randomly divided into two groups (*n* = 24): N and Abs groups. Mice in the Abs group were provided with antibiotic drinking water containing cefoxitin, gentamicin, metronidazole, and vancomycin (1 mg·mL^−1^ of each; Solarbio, China). In contrast, mice in the N group received regular drinking water. The body weight of each mouse was recorded every two days. After four weeks, the antibiotic drinking water was removed. Then, feces and mouth swabs were taken from both groups to analyze the gut and oral microbiota. The feces samples were immediately stored at −80 °C and the oral swab samples were stored in oral swab preservation solution (Sangon Biotech, China). Mice in the N and Abs groups were then divided into two groups, respectively (*n* = 12): N + N group, N + Lig group, Abs+N group, and Abs+Lig group. An experimental periodontitis model was established in the N + Lig and Abs+Lig groups. A silk ligature (5-0; Ethicon, America) was placed between the left maxillary first and second molars. Mice in the N + N and Abs+N groups had no ligatures. After two weeks, feces and mouth swabs in each group were collected before all the mice were euthanized to collect the maxillae and intestinal (ileum, cecum, and colon) tissue. The final sample size in Abs+Lig, N + Lig, Abs+N, and N + N groups are 11, 9, 11, 11 mice.

Another 30 mice were also provided with antibiotic drinking water for four weeks, the same as the Abs group. Then, the antibiotic water was replaced with regular drinking water and the mice were divided into two groups: FMT-N and FMT-Abs. The fecal microbiota of mice in the N + N and Abs+N groups were transferred to the FMT-N and FMT-Abs groups, respectively. Two weeks after FMT, feces and mouth swabs were collected in both groups and all mice received a ligature. After another two weeks, feces and mouth swabs were collected again before all the mice were euthanized to collect the maxillae and intestinal (ileum, cecum, and colon) tissue. The final sample size in FMT-N + Lig and FMT-Abs+Lig groups is 12 and 15 mice.

The animal experiments were permitted by the Ethics Committee of the Stomatological Hospital, Chongqing Medical University, Chongqing, China (approval number: CQHS-REC-2022 LSNo.003).

### Fecal microbiota transplantation

The feces of mice in the N + N and Abs+N groups were vortex mixed, respectively, with cysteine (Solarbio, China) and Na2S (Solarbio, China) in corresponding centrifuge tubes (500 mg feces, 2.5 mg cysteine, and 1 mg Na_2_S in 3 mL bacteria-free PBS). Then, the mixture for each group was centrifuged (500 r·min^−1^, 1 min) and the supernatant solution was collected. Mice in the FMT-N and FMT-Abs groups received intragastric administration of the supernatant solution from the N + N and Abs+N groups, respectively, (100 μL per mouse).

### 16S rRNA gene sequences and DNA analysis

The feces samples and oral swabs were processed by Shanghai Majorbio Bio-Pharm Technology Co. Ltd (Shanghai, China). A NanoDrop2000 (Thermo Scientific, America) was used to assess the concentration. And agarose gel electrophoresis was used to estimate the quality of DNA. The V4-V5 region of the 16S rRNA gene sequences was amplified with primers 338 F (5′-ACTCCTACGGGAGGCAGCAG-3′) and 806 R (5′-GGACTACHVGGGTWTCTAAT-3′). The amplicons were then extracted from agarose gels and purified amplicons were pooled in equimolar amounts and subjected to sequencing on an Illumina MiSeq platform.

### Micro-CT analysis of alveolar bone

Maxillae from each group were scanned using micro-CT (Skyscan1172, Skyscan, Belgium). Amira software (Amira 6.0.1, Germany) was used for three-dimensional reconstruction and histomorphometric analysis. The alveolar bone crest (ABC) to the cementoenamel junction (CEJ) was measured to analyze alveolar bone resorption.

### Histologic analyses of alveolar bone and intestinal tissue

Maxillae of mice were decalcified in 19% EDTA for 30 days. Then, 6-μm slides of maxillae and intestinal tissue were prepared for follow-up staining. An alcin-blue and periodic acid-schiff staining kit (Bioss, China) was used for colon slides. A HE staining kit (Solarbio, China) was used for maxillae and intestine slides to examine alveolar bone loss and intestinal damage. The distance between the CEJ and ABC was measured (three times/slide) to analyze alveolar bone loss. According to the protocol of Yuan et al.^50^, six regions of interest (ROI, 80 μm × 80 μm) from the interdental area of each slide were chosen for counting neutrophils, and the overall average amount was recorded. The results were then reported as cells per area of interest. Intestinal damage was evaluated according to the study by Ulrike Erben et al.^51^ A TRAP staining kit (Nanjing Jianchen Bioengineering Institute, China) was used to examine osteoclasts. Each slide was counted three times. IL-17A, IL-6, Foxp3, and IL-10 were examined by immunohistochemical staining. Maxillae slides were blocked in 10% goat serum and incubated with primary antibodies against IL-17A, IL-6, Foxp3, or IL-10 (1:400) (Bioss, China) at 4 °C overnight. Then, they were stained with secondary antibodies (Bioss, China). A semi-quantitative method was used to analyze the positive expression using Image-Pro Plus 6.0. The results were then presented as the mean integrated optical density. A slide scanner (VS200, Olympus, Japan) was used to collect images of the slides mentioned above.

### Statistical analysis

SPSS software (SPSS 19.0, America) was used in this study to analyze all the data. The data were analyzed by one-way analysis of variance and Student’s *t* tests. Significant differences were denoted at *P* < 0.05.

## Supplementary information

Supplemental Material

## Data Availability

Original data are available from the corresponding author upon reasonable request.
