# Supplementary material for: Systemic antibiotics increase microbiota pathogenicity and oral bone loss
Source: Int J Oral Sci. 2023 Jan 12;15:4. doi: 10.1038/s41368-022-00212-1 (PMC9834248; doi:10.1038/s41368-022-00212-1)
Supplement: Supplementary file 1 — Supplemental Material [file 41368_2022_212_MOESM1_ESM.docx]

**Title: Systemic antibiotics increase microbiota pathogenicity and oral bone loss**

Authors: Xulei Yuan, Fuyuan Zhou, He Wang, Xinxin Xu, Shihan Xu, Chuangwei Zhang, Yanan Zhang, Miao Lu, Yang Zhang, Mengjiao Zhou, Han Li, Ximu Zhang, Tingwei Zhang* and Jinlin Song*

**Supplemental Appendix**


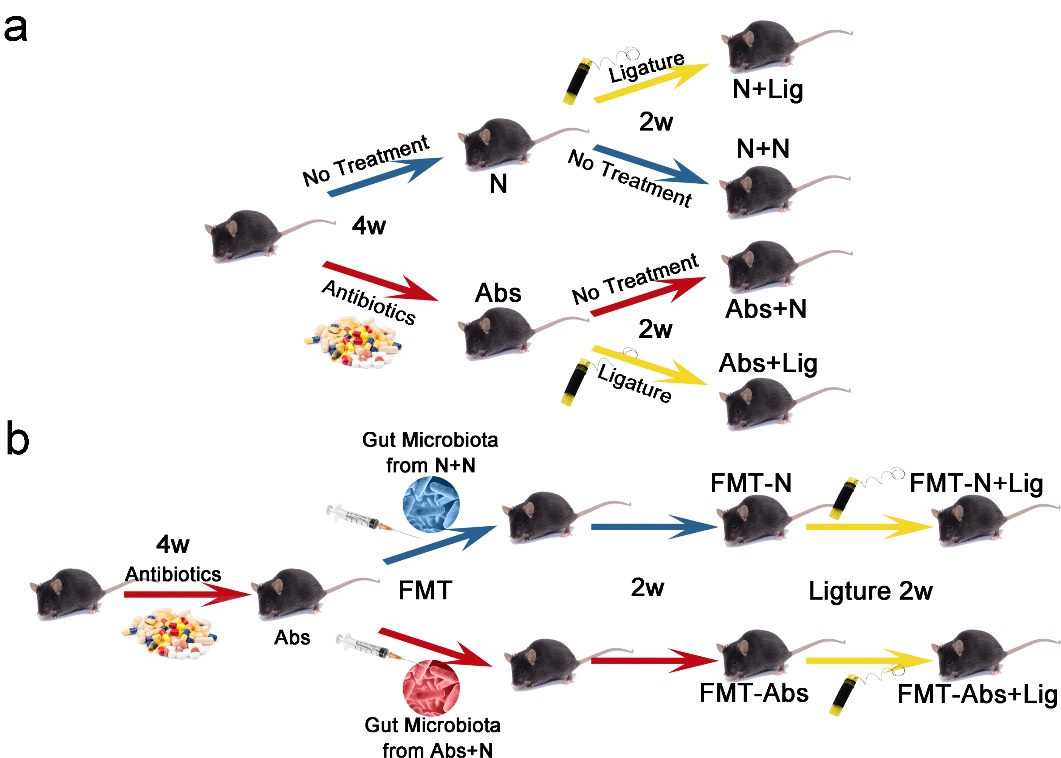


**Fig S1.** Schematic of the experiment.


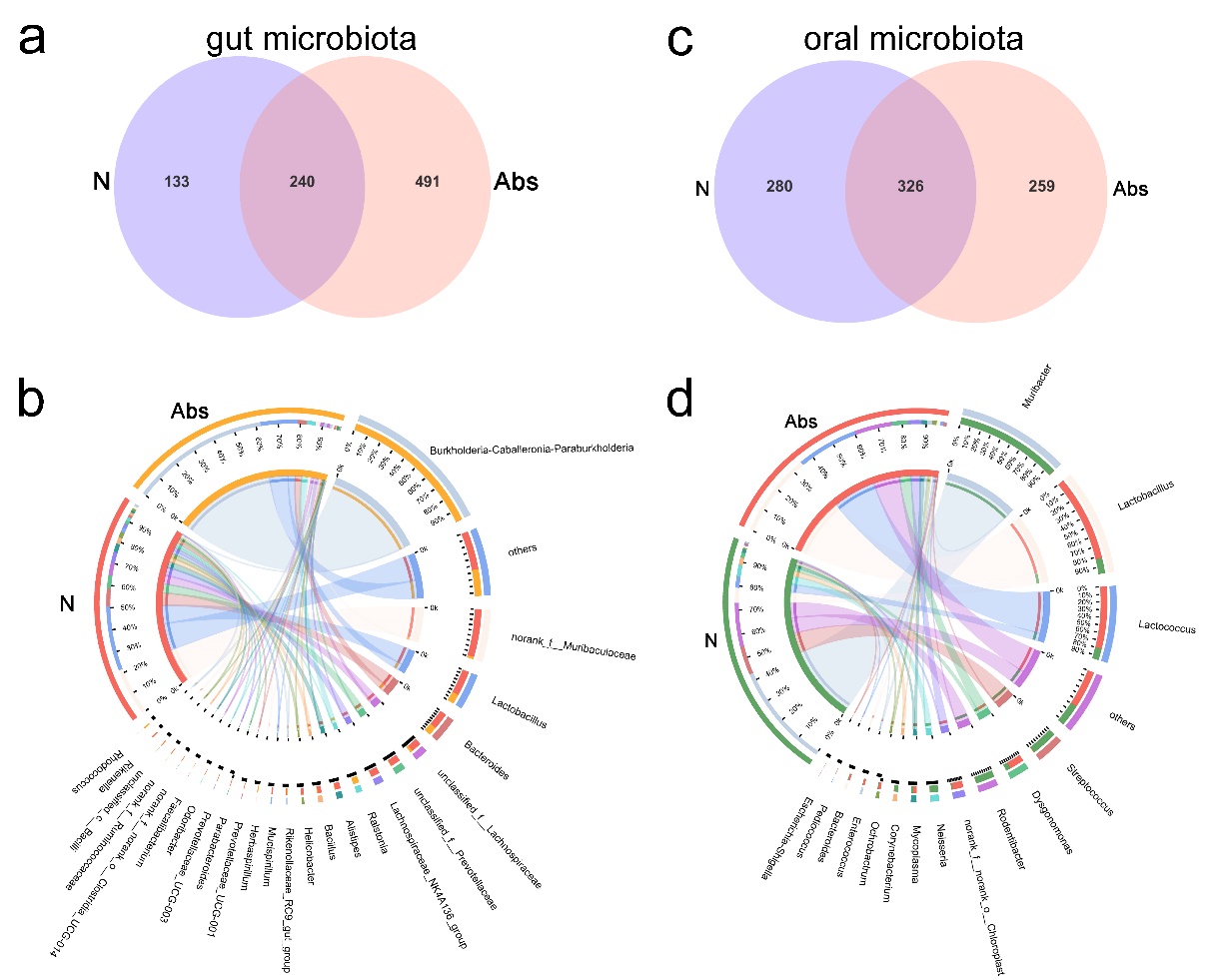


**Fig S2.** Abs and N groups: Venn diagrams of the gut (**a**) and oral (**c**) microbiota at the species level. Community Circus diagrams of the gut (**b**) and oral (**d**) microbiota at the genus level.


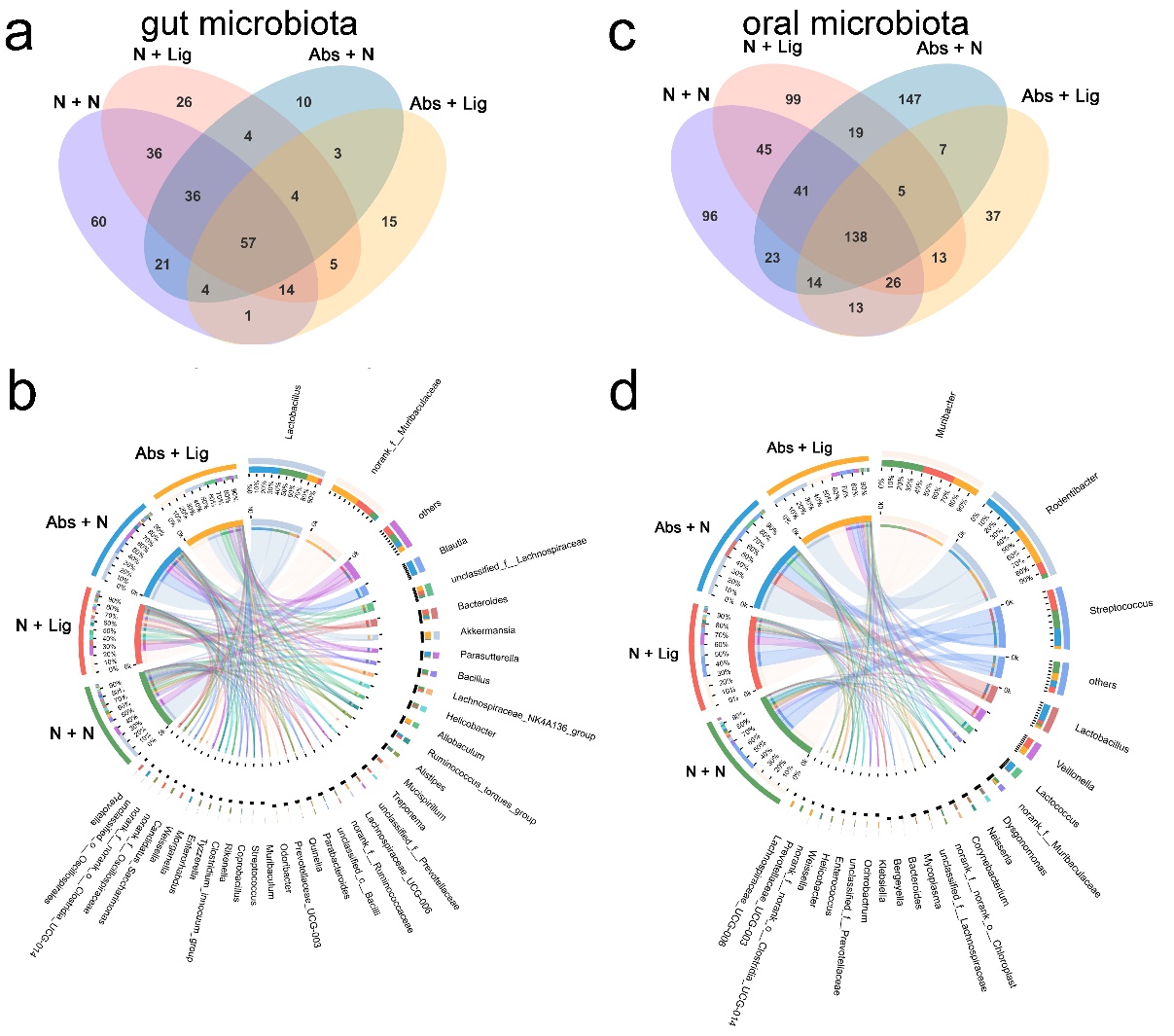


**Fig S3.** Abs+Lig, Abs+N, N+Lig and N+N groups: Venn diagrams of the gut (**a**) and oral (**c**) microbiota at the species level. Community Circus diagrams of the gut (**b**) and oral (**d**) microbiota at the genus level.


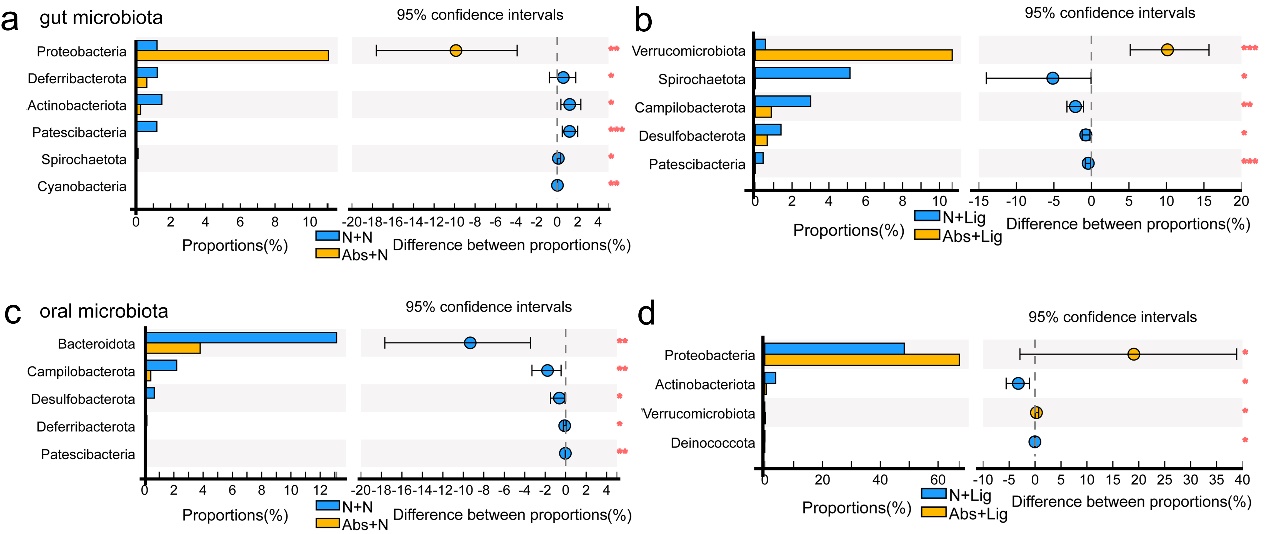


**Fig S4.** Community bar plot and Wilcoxon rank-sum test bar plot of the gut (**a-b**) and oral microbiota (**c-d**) at the phylum level.


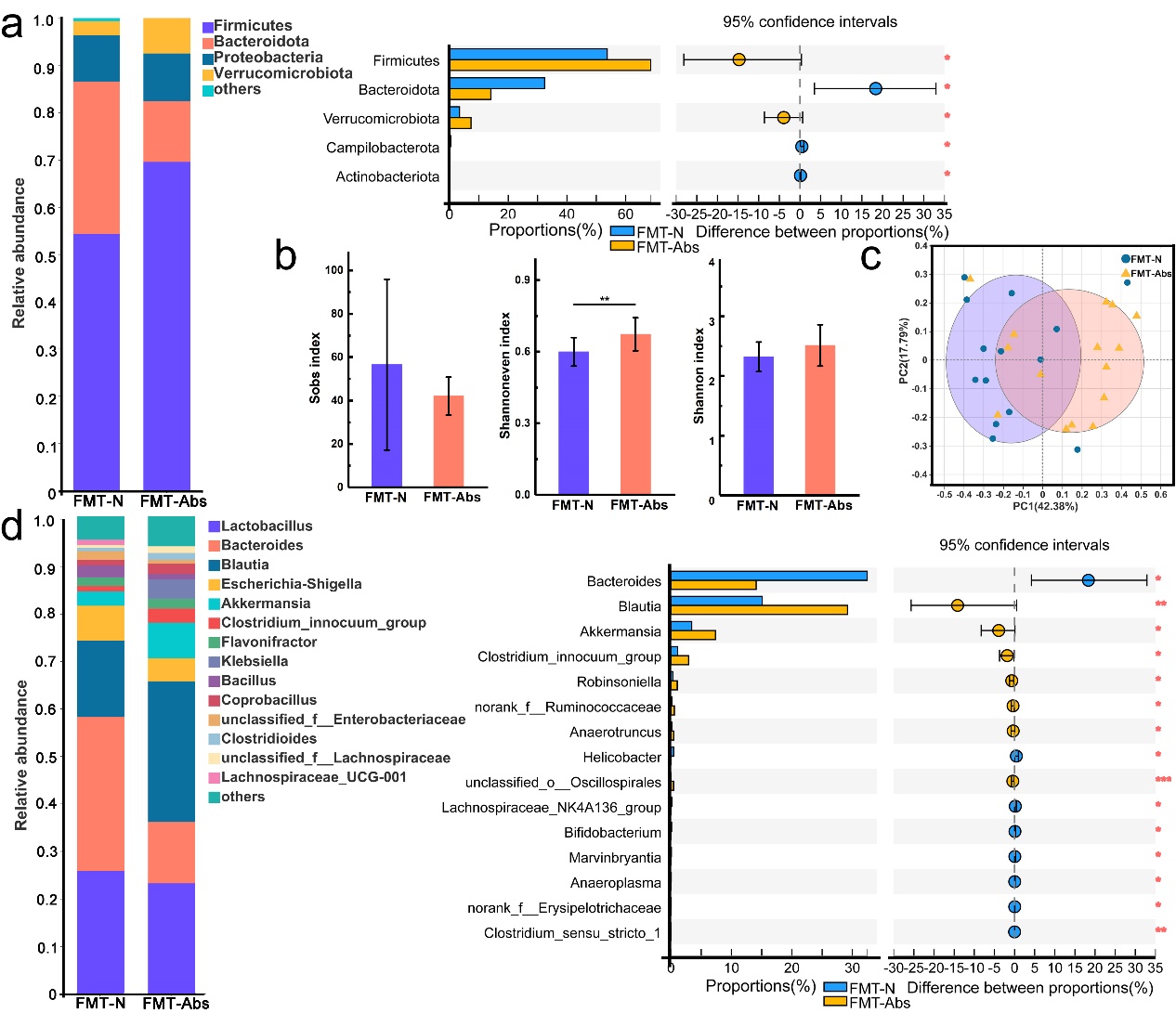


**Fig S5.** **FMT with normal mice feces improved the gut dysbiosis caused by antibiotics.** Community bar plot and Wilcoxon rank-sum test bar plot of the gut microbiota at the phylum (**a**) and genus level (**d**). Alpha diversity of the gut microbiota (**b**). PCoA analysis of the gut microbiota (**c**).

* P < 0.05, ** P < 0.01, *** P < 0.001


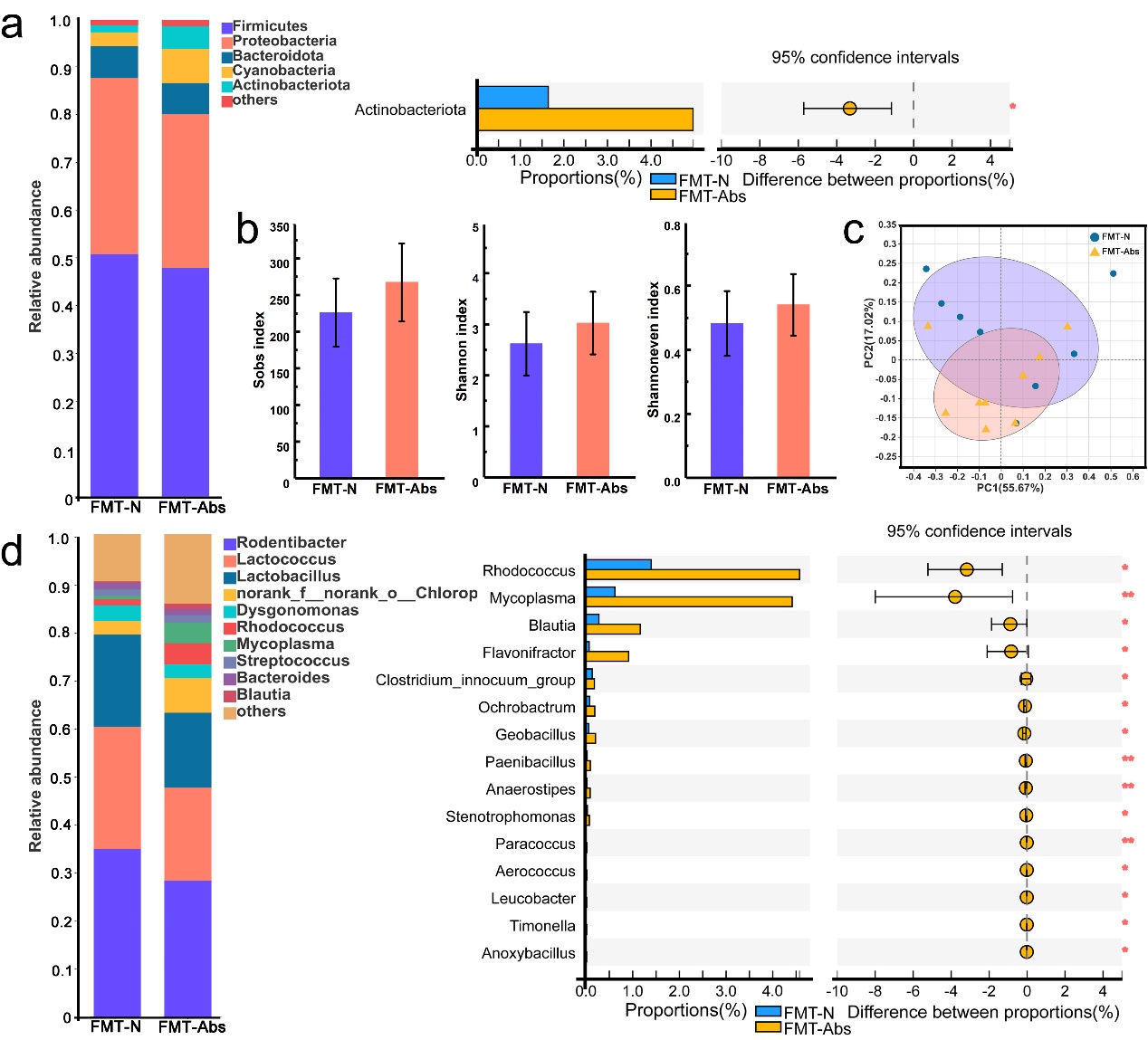


**Fig S6.** **FMT with normal mice feces had no obvious effect on the oral microbiota.** Community bar plot and Wilcoxon rank-sum test bar plot of the oral microbiota at the phylum (**a**) and genus level (**d**). Alpha diversity of the oral microbiota (**b**). PCoA analysis of the oral microbiota (**c**).

* P < 0.05, ** P < 0.01, *** P < 0.001

**
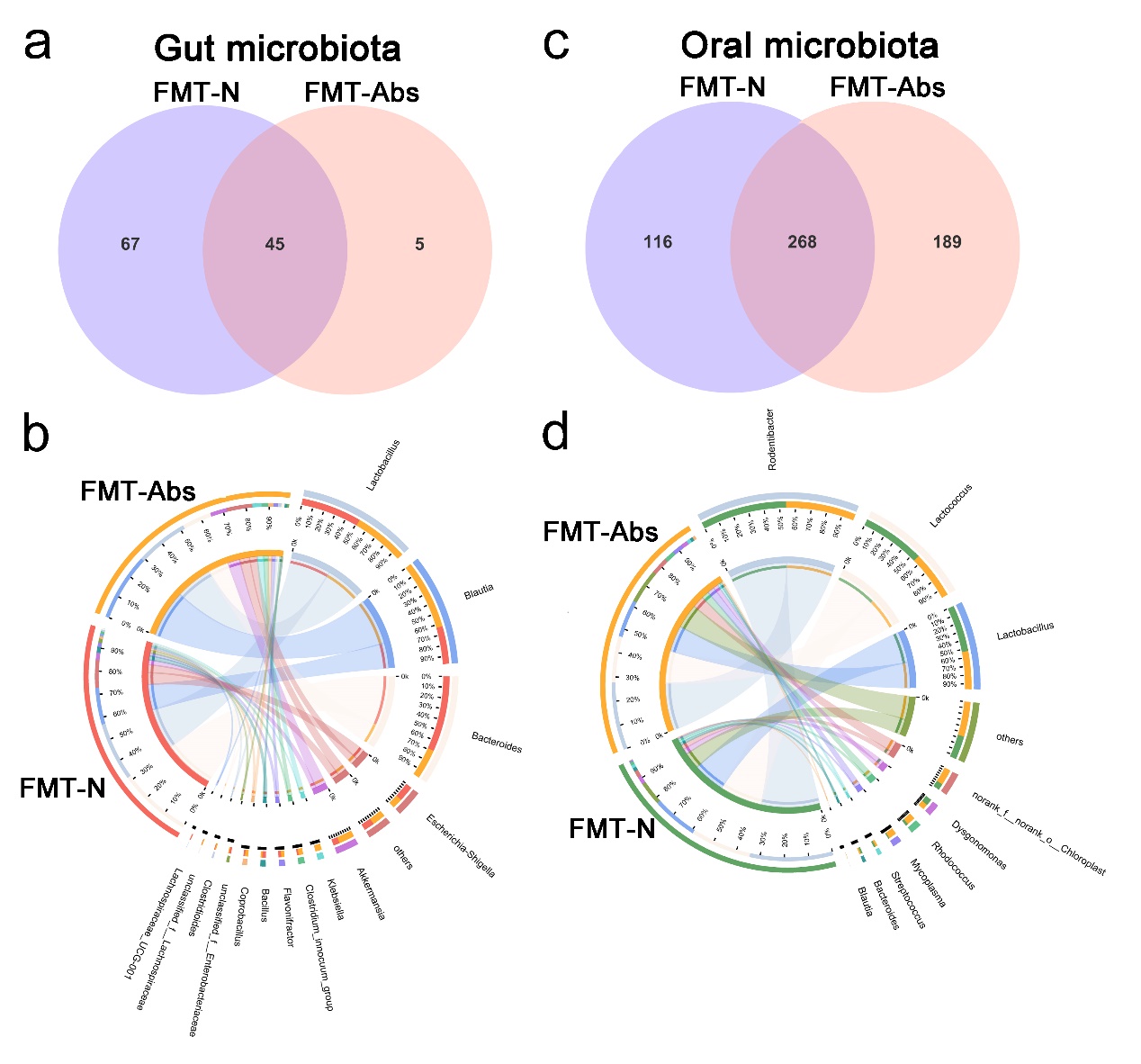
**

**Fig S7.** FMT-N and FMT-Abs groups: Venn diagrams of the gut (**a**) and oral (**c**) microbiota at the species level. Community Circus diagrams of the gut (**b**) and oral (**d**) microbiota at the genus level.


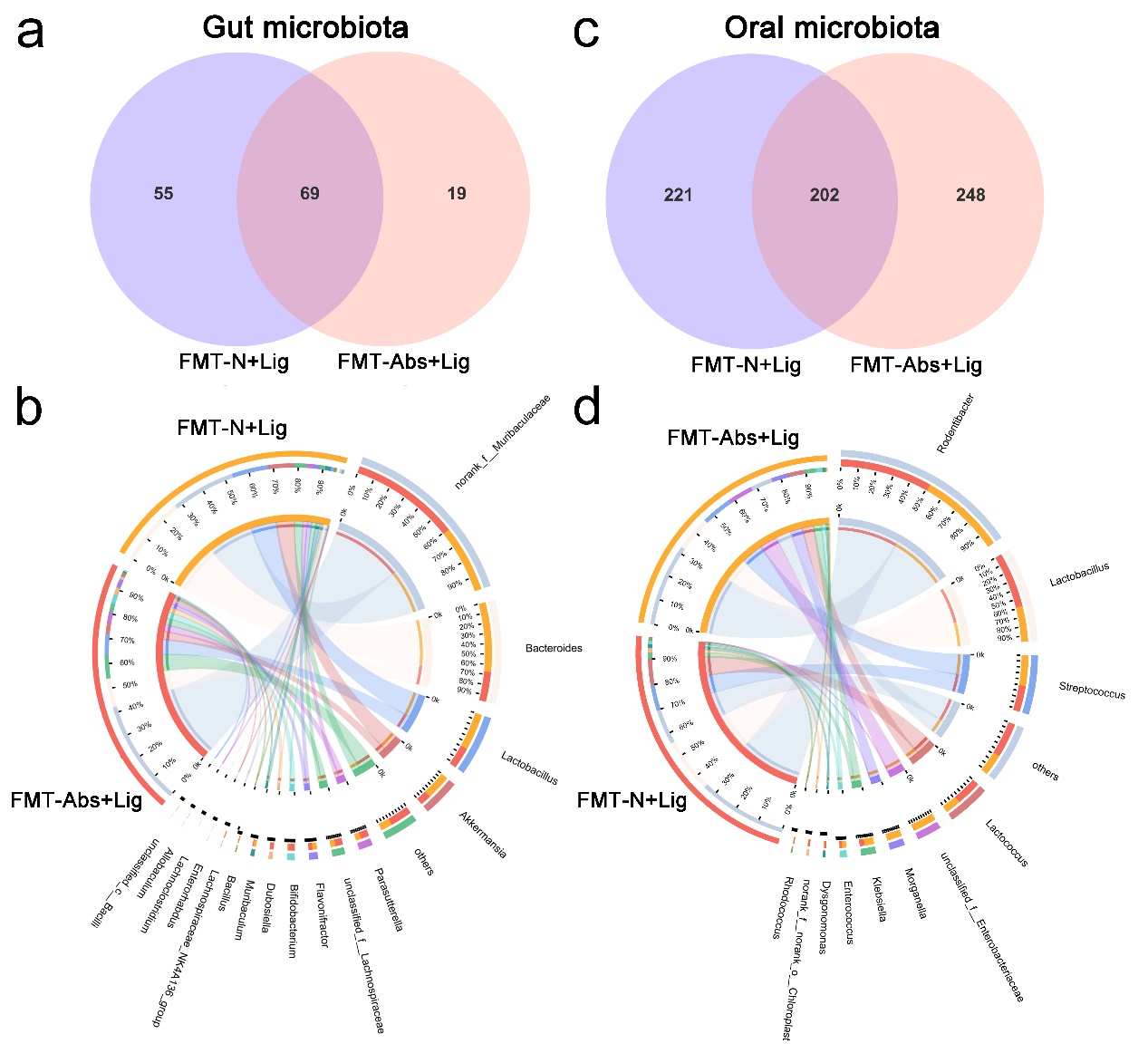


**Fig S8**. FMT-N+Lig and FMT-Abs+Lig groups: Venn diagrams of the gut (**a**) and oral (**c**) microbiota at the species level. Community Circus diagrams of the gut (**b**) and oral (**d**) microbiota at the genus level.
